# Trait Energy and Fatigue May Be Connected to Gut Bacteria among Young Physically Active Adults: An Exploratory Study

**DOI:** 10.3390/nu14030466

**Published:** 2022-01-21

**Authors:** Ali Boolani, Karyn M. Gallivan, Kristin S. Ondrak, Courtney J. Christopher, Hector F. Castro, Shawn R. Campagna, Christopher M. Taylor, Meng Luo, Scot E. Dowd, Matthew Lee Smith, Lauri O. Byerley

**Affiliations:** 1 Department of Physical Therapy, Clarkson University, Potsdam, NY 13699, USA; 2 Department of Biology, Clarkson University, Potsdam, NY 13699, USA; 3 Sports and Health Sciences, School of Health Sciences, American Public University System, Charles Town, WV 25414, USA; kgallivan@apus.edu (K.M.G.); kristin.ondrak@mycampus.apus.edu (K.S.O.); 4 Department of Chemistry, University of Tennessee, Knoxville, TN 37996, USA; cleathe3@vols.utk.edu (C.J.C.); hcastrog@utk.edu (H.F.C.); campagna@utk.edu (S.R.C.); 5 Biological and Small Molecule Mass Spectrometry Core, University of Tennessee, Knoxville, TN 37996, USA; 6 Department of Microbiology, Immunology and Parasitology, Louisiana State University Health Sciences Center, New Orleans, LA 70112, USA; ctay15@lsuhsc.edu (C.M.T.); mluo2@lsuhsc.edu (M.L.); 7 Molecular Research LP, 503 Clovis Rd, Shallowater, TX 79363, USA; sdowd@mrdnalab.com; 8 Department of Environmental and Occupational Health, School of Public Health, Texas A&M University, College Station, TX 37916, USA; matthew.smith@tamu.edu; 9 Center for Population Health and Aging, Texas A&M University, College Station, TX 77807, USA; 10 Department of Physiology, Louisiana State University Health Sciences Center, New Orleans, LA 70112, USA

**Keywords:** trait mental fatigue and energy, trait physical fatigue and energy, gut microbiome, gut microbiota

## Abstract

Recent scientific evidence suggests that traits energy and fatigue are two unique unipolar moods with distinct mental and physical components. This exploratory study investigated the correlation between mental energy (ME), mental fatigue (MF), physical energy (PE), physical fatigue (PF), and the gut microbiome. The four moods were assessed by survey, and the gut microbiome and metabolome were determined from 16 S rRNA analysis and untargeted metabolomics analysis, respectively. Twenty subjects who were 31 ± 5 y, physically active, and not obese (26.4 ± 4.4 kg/m^2^) participated. Bacteroidetes (45%), the most prominent phyla, was only negatively correlated with PF. The second most predominant and butyrate-producing phyla, Firmicutes (43%), had members that correlated with each trait. However, the bacteria *Anaerostipes* was positively correlated with ME (0.048, *p* = 0.032) and negatively with MF (−0.532, *p* = 0.016) and PF (−0.448, *p* = 0.048), respectively. Diet influences the gut microbiota composition, and only one food group, processed meat, was correlated with the four moods—positively with MF (0.538, *p* = 0.014) and PF (0.513, *p* = 0.021) and negatively with ME (−0.790, *p* < 0.001) and PE (−0.478, *p* = 0.021). Only the Firmicutes genus *Holdemania* was correlated with processed meat (r = 0.488, *p* = 0.029). Distinct metabolic profiles were observed, yet these profiles were not significantly correlated with the traits. Study findings suggest that energy and fatigue are unique traits that could be defined by distinct bacterial communities not driven by diet. Larger studies are needed to confirm these exploratory findings.

## 1. Introduction

Approximately 45% of the United States (US) population experiences elevated and persistent fatigue, a common, costly, and poorly understood problem [1,2,3]. It has been estimated that fatigue costs employers over $136 billion per year in lost productivity [4]; however, these estimates do not account for fatigue-related driving and other accidents [5,6], poor medical performance [7], school absences [8,9], and declines in school performance and negative health outcomes [9]. Fatigue is underreported in medical care [10] and linked to many diseases and disorders [3]. Despite fatigue’s high financial and social costs, it is a poorly understood problem despite there being over 250 different instruments and no consensus about how best to measure fatigue [11]. One challenge for fatigue researchers is articulating the conceptual relationship between fatigue and energy.

Until recently, both colloquially and in research, feelings of fatigue were usually defined as “a lack of energy,” suggesting that energy and fatigue were opposite ends of the same continuum. However, recent evidence suggests that energy and fatigue are two unique unipolar moods with distinct neurohormonal [12] and physiological [13,14] correlations. Further complicating this matter is evidence that suggests there are distinct mental and physical components of energy and fatigue (e.g., there are four moods, mental energy (ME), mental fatigue (MF), physical energy (PE), and physical fatigue (PF)) [15,16,17], and recent literature makes an additional distinction between the concepts of state and trait mental and physical energy and fatigue. As is the case with all moods, the state aspect of energy and fatigue are considered transient (short-term) and can be influenced by outside factors such as physical activity [18,19], sleep [20], and certain foods [21,22]. Recent evidence suggests that there is a trait aspect of energy and fatigue [15,16,23], which modifies the effects of various interventions on state energy and fatigue [15,23], objective measures of energy and fatigue [23], fine motor control [23], and other moods such as anxiety and depression [23].

In 2018, Loy and colleagues [12] provided evidence that state energy was associated with dopamine, while state fatigue was associated with serotonin, histamine, and inflammatory cytokines. Subsequently, Boolani and colleagues [13] provided cross-sectional evidence that peripheral mitochondrial function and normalized resting metabolic rate (nRMR) were associated with feelings of state energy; however, they did not report any physiological associations with feelings of state fatigue. Dupree and colleagues [14], in a case-controlled interventional study, reported changes in salivary Annexin A1 with feelings of ME, but no changes in salivary biomarkers were reported with feelings of MF. In 2017, Eshragh and colleagues [24] reported unique and overlapping epigenetic associations between feelings of energy and fatigue measured over two-time points. While this study [24] measured state energy and fatigue, as participants were asked how they felt in the moment, the stability of the measures over 6 months suggests that the study actually captured the trait aspect of energy and fatigue. However, these researchers [24] did not explicitly use a trait measure of energy and fatigue and, additionally, they did not differentiate between the mental and physical aspects of energy and fatigue. All of these studies suggest there may be unique yet overlapping biologic correlates of trait energy and fatigue that require further examination.

The human gastrointestinal tract contains thousands of bacterial species, primarily anaerobes, from two predominant phyla, Firmicutes and Bacteroidetes [25,26]. Most are located in the large bowel, where they ferment nondigestible food, making available nutrients and other substrates, like the short-chain fatty acid butyrate [27,28]. These processes, among others, performed by the gut microbiota are essential for maintaining homeostasis and normal gut physiology [29]. Several diseases have been linked to gut microbiota dysbiosis, such as obesity, coronary heart disease, diabetes, and inflammatory bowel disease [30]. The gut microbiota has also been implicated in mental health and cognition, and the existence of the gut-brain axis is well established [31].

Diet is one of the predominant influencers of gut microbiota composition. It determines the relative abundance of many microbial species, and these changes in microbial composition influence metabolic processes, and subsequently, the metabolome [32,33]. For example, long-term dietary patterns, particularly the intake of protein and animal fat (Bacteroides) versus carbohydrates or plant-based foods (Prevotella), are associated with so-called enterotypes [34]. Plants are rich in fiber and anti-inflammatory compounds, like polyphenols, which enter the colon where colonic microbiota convert them to bioavailable and biologically active compounds (e.g., apples) [35]. Evidence suggests that consuming polyphenol-rich diets may be associated with increased feelings of mental energy [16,36]. In addition, Mediterranean diets have been known to reduce fatigue; however, the authors did not differentiate between mental and physical fatigue [37]. Taken together, it may be hypothesized that diets associated with increased feelings of energy or reduced feelings of fatigue may also be associated with gut microbiome changes.

Recently, several studies reported an association between gut microbiome and feelings of fatigue [38,39,40,41]; however, these studies did not distinguish between the mental and physical aspects of fatigue. These preliminary findings have reported reduced diversity and altered gut microbiome among patients with cancer-related fatigue [38,40] and myalgic encephalomyelitis/chronic fatigue syndrome (ME/CFS) [39,41]. These studies were exploratory in nature, with some studies not adjusting analyses to account for multiple comparisons [38,40,41] and others comparing ME/CFS patients to healthy control populations [39,41]. One limitation of these recent studies is that findings have cannot be extrapolated to healthy populations. Another limitation is that previous studies [38,39,40,41] have measured fatigue as a lack of energy rather than measuring energy and fatigue as two separate unipolar moods with their own unique mental and physical components. Taken together, these limitations suggest there is a need to examine the association between gut microbiome diversity and feelings of mental and physical energy and fatigue uniquely among healthy individuals. Due to the limitations of current literature and the costs of performing these analyses, it is advisable to perform an exploratory study that may provide researchers with interesting findings without worrying about false positives to guide future targeted studies [42]. Therefore, this exploratory study will uniquely add to the literature by documenting the gut microbiota that may be correlated with the four distinct trait aspects of mental energy (ME), mental fatigue (MF), physical energy (PE), and physical fatigue (PF).

## 2. Materials and Methods

### 2.1. Subjects

Subjects were recruited from thirty-nine individuals who previously participated in a study investigating the gut microbiome. The potential subjects were contacted by email and invited to complete a brief survey about traits mental and physical energy and fatigue. The email described the study and explained that their survey responses would be correlated with their fecal microbiome. Individuals were excluded if they met any of the following criteria: (1) took an antibiotic over the last three months; (2) consumed an anti-diarrhea medicine in the last week; (3) took a laxative in the last week; (4) consumed prebiotics in the last week; (5) consumed probiotics in the last week; (6) been diagnosed with cancer; (7) been diagnosed with Crohn’s disease; (8) taking prescription medications other than oral contraceptives; (9) cutting weight for an upcoming competition; (10) under the age of 25 years; or (11) lived outside the contiguous United States. Informed consent was obtained from all subjects involved in the study. A total of 20 subjects consented and completed the survey. All study procedures were approved by the Institutional Review Board at the American Public University System (2021–045–OL, 14 April 2021).

### 2.2. Survey

The survey was conducted online using Qualtrics (Qualtrics, XM, Provo, UT, USA). The mental and physical state and trait energy and fatigue scales [18] were used to discriminate between mental and physical energy and fatigue. The reliability and temporal stability of these scales has been previously demonstrated [21,22,23,43,44,45,46,47]. For the current study, reliability was tested using Cronbach’s Alpha test in SPSS (IBM Corp. Released 2020. IBM SPSS Statistics for Windows, Version 27.0. Armonk, NY, USA: IBM Corp). The scores were PE, 0.767, PF, 0.899, ME, 0.890, and MF, 0.893.

### 2.3. Diet Recall

The subjects were asked to recall the foods they ate the 24 h prior to collecting their fecal sample using an automated, web-based, self-administered 24 h dietary assessment (ASA24) (https://epi.grants.cancer.gov/asa24/) (accessed on 1 January 2022) [48]. The program is freely available and can be accessed on the internet and mobile devices.

Fecal sample collection and DNA isolation: Each subject participated in an earlier study in which a stool sample was collected, the DNA was isolated, and the bacteria DNA was analyzed to identify the microbes present. Briefly, for that study, the DNA was extracted using the QIAamp DNA Stool Mini Kits (Qiagen, Germantown, MD, USA) modified to include bead-beating and RNase A treatment. A negative control was set for checking any potential bacterial DNA existing in chemicals or involved during the DNA extraction process. Purity and quantity were determined using a Thermo Scientific™ NanoDrop™ spectrophotometer (ThermoFisher Scientific, Waltham, MA, USA).

### 2.4. Microbial Community Analysis

Two amplification steps were performed to prepare a sequencing library using the AccuPrime Taq high-fidelity DNA polymerase system (Invitrogen, Carlsbad, CA, USA). A negative control with the control from DNA extraction and a positive control of Microbial Mock Community HM-276D (BEI Resources, Manassas, VA, USA) were set during amplicon library preparation. Next, 16S ribosomal DNA hypervariable region V4 was amplified using genomic DNA and the gene-specific primers with Illumina adaptors: Forward 5’TCGTCGGCAGCGTCAGATGTGTATAAGAGACAG GTGCCAGCMGCCGCGGTAA3’; Reverse 5’ GTCTCGTGGGCTCGGAGATGTGTATAAGAGACAG GGACTACHVGGGTWTCTAAT 3’. Polymerase chain reaction (PCR) products were purified using AMPure XP beads, and the purified amplicon DNA was amplified using the primers with different molecular barcodes: forward 5’ AATGATACGGCGACCACCGAGATCTACAC [i5] TCGTCGGCAGCGTC 3’; reverse 5’ CAAGCAGAAGACGGCATACGAGAT [i7] GTCTCGTGGGCTCGG 3’. The indexed amplicon libraries purified using AMPure XP beads and quantified using Quant-iT PicoGreen (Invitrogen) were normalized and pooled. The pooled library was quantified using KAPA Library Quantification Kit (Kapa Biosystems), diluted, and denatured according to Illumina’s sequencing library preparation guidelines. In addition, 10% PhiX was added to the sequencing library as an internal control and increased 16S RNA amplicon library diversity. The paired-end sequencing was performed on an Illumina MiSeq (Illumina, San Diego, CA, USA) using the 2 × 250 bp V2 sequencing kit.

Raw fastq files were processed using QIIME2 with the DADA2 plugin [49]. Forward and reverse reads were truncated to a uniform length of 240 bp, and 20 bp were trimmed off of the front of each read to remove the primer. Amplicon sequence variants (ASVs) identified by DADA2 were merged, and any that fell out of the expected 250–255 bp length were discarded. Contingency-based filtering was performed to remove any ASVs that appeared in only one sample, and the consensus method was used to remove chimeric ASVs. A phylogenetic tree for diversity analysis was built by aligning remaining ASVs using mafft [50] and fasttree [51]. Taxonomic classification was performed using Greengenes v13.8 [52]. After primary data analysis, the remaining reads were analyzed using QIIME2 (Quantitative Insights Into Microbial Ecology) [53].

Read counts ranged from 14,628 to 90,465, with an average read count per sample of 56,041. Alpha rarefaction was performed at a level of 14,600 reads.

Prediction of Metabolic Profile: Potential microbial functions were identified from the 16S sequencing data. The raw data were formatted and imported into QIIME2. The dereplicated feature table and representative sequences were then used for closed-reference clustering against the Greengenes 13_5 97% OTUs reference database. The closed-reference OTU table was used as input into the PICRUSt [54] pipeline, and the resulting PICRUSt metagenome data were further analyzed using STAMP (Statistical Analysis of Metagenomic Profiles) [55] using pathways labeled at Level 2.

### 2.5. Metabolomics

Samples from the 20 survey subjects were processed at the Biological and Small Molecule Mass Spectrometry Core (BSMMSC), University of Tennessee, Knoxville, TN, USA (RRID: SCR_021368). Samples were pre-weighed (~50 mg aliquots) and extracted in biological triplicate. Briefly, water-soluble metabolites were extracted from fecal samples using an acidic acetonitrile extraction procedure [56]. An untargeted metabolomics method was employed to analyze the fecal microbiome using ultra high-performance liquid chromatography coupled to high-resolution mass spectrometry (UHPLC-HRMS). A 25 min method using a water:methanol solvent system with tributylamine as an ion-pairing reagent was used for reverse-phase chromatographic separation. This was accomplished by using a Synergi 2.6 µm Hydro RP column (100 mm × 2.1 mm, 100 Å; Phenomenex, Torrance, CA, USA) and an UltiMate 3000 pump (Dionex). Eluted analytes were then ionized via negative mode electrospray ionization, and mass spectral analysis was accomplished using a Thermo Scientific Exactive Plus Orbitrap (San Jose, CA, USA) operating in full-scan mode [57,58]. Raw spectral files were converted to mzML files using the msCovert package from ProteoWizard [59]. Metabolites were identified manually by exact mass (±5 ppm) and retention time using an in-house library of metabolites and the open-source software, metabolomics analysis and visualization engine (MAVEN) [60,61]. There were 170 identified metabolites from the untargeted metabolomics analysis.

### 2.6. Statistical Analysis

Microsoft Excel (Office 365) and SPSS (IBM Corp. Released 2020. IBM SPSS Statistics for Windows, Version 27.0. Armonk, NY, USA: IBM Corp) were used for data analyses. Variables were evaluated for normality of distribution using a combination of histograms and the Shapiro-Wilks tests for normality. Neither the trait variables nor the gut microbiome data were normally distributed. Trait fatigue (both mental and physical) was positively skewed, while trait mental energy (both mental and physical) was negatively skewed. This skewness is similar to what has been reported in previous studies using these constructs [15,23]. Exponential, power, arscine, and logarithmic transformation techniques were attempted for all skewed variables; however, these transformations did not result in normally distributed data (*p* > 0.05), and the histograms did not differ much from the original. Thus, non-parametric analyses were used. Data were expressed as medians and interquartile range, and the actual p-values were reported. Relationships with *p* < 0.05 were considered statistically significant. Spearman’s rho coefficients were calculated to determine correlations between each trait (MF, ME, PF, and PE), bacteria species, diversity, predicted functional pathways, dietary nutrients, and food groups. Additionally, Spearman’s rho was used to identify the correlation between the bacterial species and the traits, as well as the correlation between the nutrients and food groups correlated with the traits. Cronbach’s Alpha coefficients were computed to test reliability of the trait measures.

Metabolomics spectral data were normalized by weight for each sample. The peak intensities were normalized by the weight of the fecal matter used in each aliquot (~50–120 mg) [62,63]. The normalized data were used for principal component analysis (PCA), an unsupervised dimensionality reduction tool. MetaboAnalyst 5.0 was used to generate PCA plots, where the normalized data were filtered by interquartile range, log-transformed, and Pareto scaled [64]. The PCA plot partitioned the subjects into two clusters. To determine if specific traits (MF, ME, PF, PE) corresponded with each metabolic cluster, the subjects were assigned a group based on their clustering location. Normality of distribution for each trait was checked using a combination of histograms and Shapiro-Wilks tests. Data were abnormally distributed and exponential, power, arscine, and logarithmic transformation techniques were used to transform the data. None of the techniques produced normally distributed data in either group. Therefore, to determine differences in trait scores between the two clusters, Mann Whitney-U tests were used to compare the trait survey responses between each cluster.

This was an exploratory study to identify unique relationships and guide future research efforts, so unadjusted findings are reported. If the Benjamini-Hochberg False Discovery Rate (FDR) of 30% was used to correct for multiple tests post hoc, then none of the values in this study would be statistically significant.

## 3. Results

### 3.1. Subject Description

The average age of the 20 subjects (14 males, 6 females) was 31.1 ± 5.0 years. Their height, weight, and BMI were 67.7 ± 4.4 inches, 172.1 ± 33.5 lbs, and 26.4 ± 4.4 kg/m^2^, respectively. Although the mean BMI was in the overweight category, the subjects were physically active, and their primary exercise was resistance training (Total Walking MET-minutes/week 1973.4 ± 2230.4; Total Moderate MET-minutes/week total 2587.5 ± 2224.0; Total Vigorous MET-minutes/week 4196.1 ± 4069.1; Total Physical Activity MET-minutes/week, 8757.0 ± 4683.2; Sitting Total Minutes/week 2074.5 ± 925.6; Average Sitting Total Minutes/day 296.4 ± 132.2). Table A1 shows these characteristics and is presented in Appendix A.

### 3.2. Trait Measures

Trait PE was 8 ± 1.8 with a range of 3–10, and trait PF was 3.6 ± 2.6 with a range of 0–12. Trait ME was 7.25 ± 2.5 with a range of 0–10, and trait MF was 4.2 ± 2.6 with a range of 0–12. The median and interquartile ranges are shown in Table A2 presented in Appendix A.

### 3.3. Diversity Measures

Only one alpha diversity measure, Faith PD, was correlated with one trait, PF. Faith PD is the sum of the branch lengths on the phylogenetic tree connecting all members of the set. The higher the Faith PD sum, the lower the PF measure (−0.509, *p* = 0.022). These data are presented in Table A2 in Appendix A.

### 3.4. Bacteria Taxa Correlated with Four Traits

The bacteria correlated with each trait are shown in Table 1. Traits ME and PF were correlated with more than one phylum, respectively. Only trait mental energy was correlated with members of the Actinobacteria and Verrumomicrobia, while members of Proteobacteria and Bacteroidetes were only associated with trait PF. Interestingly, at least one member of the Firmicutes phyla was correlated with every trait, but only one Firmicute member, c__Clostridia;o__Clostridiales;f__Lachnospiraceae;g__Anaerostipes;s__, was correlated with more than one trait (i.e., ME, MF, PF).

### 3.5. Predicted Functional Pathways Correlated with Traits

The functional pathways correlated with each trait are shown in Table 2. Functional metabolic pathways were predicted from the bacterial gene sequences and correlated with each trait. MF and PF did not associate with any functional pathways. Only one metabolic pathway was correlated with PE, while 19 were correlated with trait ME. Of the 19, 11 (57%) were metabolism-correlated pathways, and 6 of these 11 (55%) were involved with xenobiotics biodegradation and metabolism.

### 3.6. Nutrients and Food Groups Correlated with the Four Traits

The average fiber intake was 20.6 ± 2.5 g and ranged from 3.1 g to 46.0 g. Table 3 shows the significant correlations between the four traits, two nutrients (folate and lycopene), and four food groups (i.e., total dark green, red and orange, starchy, and other vegetables; dark green vegetables; grains defined as whole grains and which contain the entire grain kernel; and frankfurters, sausages, corned beef, and luncheon meat). Folate and lycopene can be found in three of the four identified food groups (i.e., total dark green, red and orange, starchy, and other vegetables; dark green vegetables; and grains defined as whole grains and which contain the entire grain kernel). Interestingly, folate and food sources rich in folate, dark green vegetables and total dark green, red and orange, starchy, and other vegetables were positively correlated with ME. Lycopene was positively correlated with both mental and physical fatigue. Processed meats like frankfurters, sausage, corned beef, and luncheon meat correlated with all four traits; negatively with ME and PE and positively with MF and PF.

### 3.7. Nutrients and Food Groups Correlated with Bacteria Correlated with the Four Traits

Since several bacteria and food groups were correlated with at least one of the four traits, correlations were examined between the bacteria that mapped to one of the traits and the food groups correlated with at least one trait (Table 4). Interestingly, only one bacterium was correlated with each food group and most were from the Erysipelotrichi class. Only Firmicutes;c__Erysipelotrichi;o__Erysipelotrichales;f__Erysipelotrichaceae;g__Holdemania was correlated with processed meats, and, interestingly, this bacteria was negatively correlated with trait PE.

### 3.8. Metabolomics

**Figure 1:**
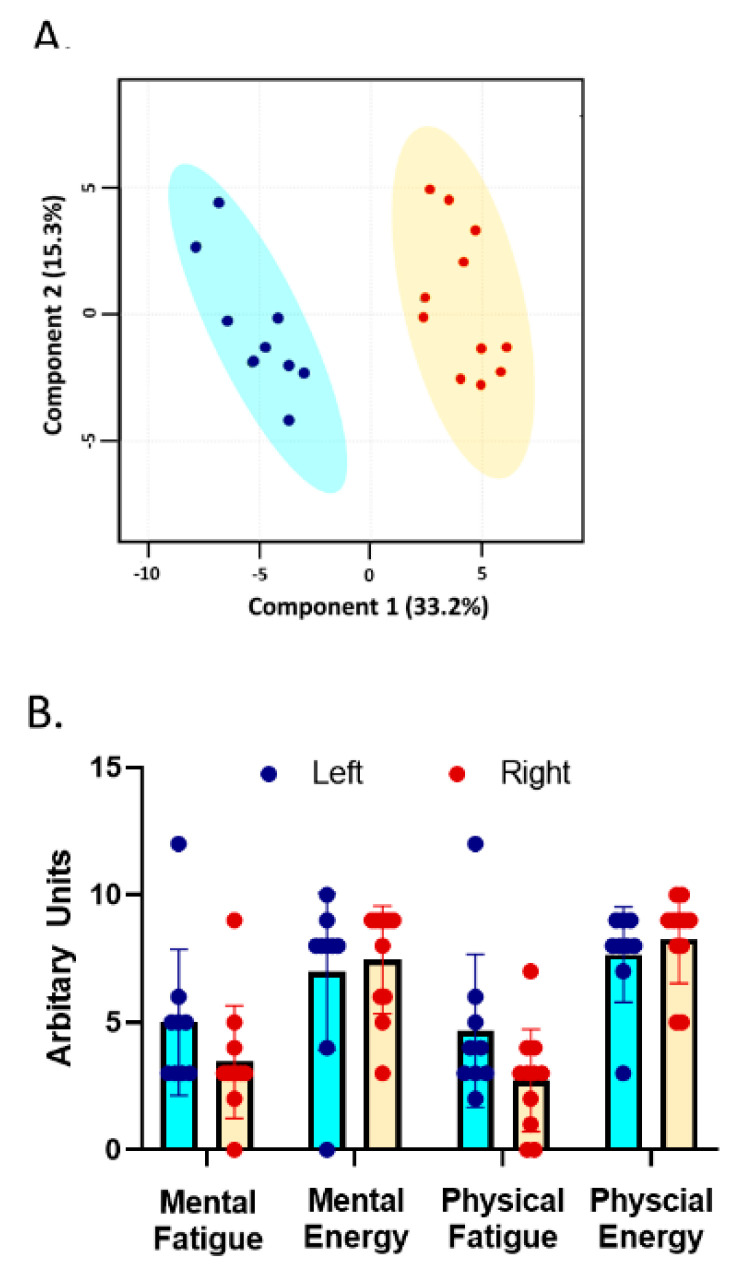
A principal component analysis (PCA) for the 20 survey subjects. (**A**) clearly shows two distinct clusters: left blue and right red. (**B**) shows the average ± SD for each trait. MF, ME, PF, and PE were not significantly different between the two clusters.

PCA analysis revealed that the subjects naturally separated into two distinct clusters (left or right, Figure 1A). Of the identified metabolites, 100 metabolites had a significantly higher relative abundance in the left group (presented in blue), including but not limited to biotin, serine, and shikimate. The right group (presented in red) had only six metabolites with a higher relative abundance, including propionyl-CoA, acetyl-CoA, and NAD+. There were no statistically significant differences in PF, PE, MF, or ME when comparing the subjects on the left to those on the right (Figure 1B). Therefore, the separation of the clusters observed on the PCA plot cannot be attributed to traits, so distinct metabolic profiles based on MF, ME, PF, and PE were not observed in the study.

## 4. Discussion

This exploratory study provides interesting evidence that gut microbiota, diet, and the corresponding metabolome may be correlated with four distinct mental and physical energy and fatigue traits. Findings provide evidence that may assist researchers when further exploring the associations between gut microbiota and trait-level mental and physical energy and fatigue. Although unique metabolic profiles were apparent in the PCA analysis, there was no evidence to attribute these differences to traits energy and fatigue. However, these findings indicate distinct bacteria may be correlated with each trait except for one, supporting previous findings that feelings of mental and physical energy and fatigue may be unique with some overlap [13,15,16]. In addition, only one bacterium was correlated with more than one trait, *Anaerostipes*, in the expected direction (i.e., positively correlated with mental energy and negatively correlated with mental and physical fatigue traits).

When examining bacterial functional pathways, none of the pathways were correlated with traits mental and physical fatigue; however, most of the pathways (22 out of 23) were correlated with trait mental energy. Most of the pathways fell under Metabolism (level 1) and Xenobiotic Biodegradation (level 2). Unfortunately, it is not apparent why more of these pathways were expressed in trait mental energy, which warrants further investigation. The metabolic pathway for bacterial invasion of epithelial cells was negatively correlated with trait physical energy, and again, it is not clear what this means and needs further investigation.

Only the Faith PD, a measure of alpha diversity, was negatively correlated with trait physical fatigue, suggesting that individuals who report normally feeling physically fatigued also had the lowest diversity of bacteria. The American Gut Project found that the gut microbiome is more diverse in people who eat more than 30 types of plants a week [65]. Plants are rich in fiber compared with animal-derived foods, and the Western diet, typically eaten in the USA, is high in animal protein and low in plant sources. Subjects in this study self-identified being physically active and often participating in resistance training. Their average protein intake was 136.2 ± 15.3 g/day (32.2 minimum and 272 maximum). Subjects’ fiber intake ranged from a low of 3 g/day to 46 g/day, and the average intake was less than the daily recommended intake (DRI) for males (38 g/day) and females (25 g/day) [66]. Only six subjects met or exceeded the DRI. For this sample, it is possible that the inadequate intake of fiber reduced the alpha diversity of their gut microbiome.

Findings from this study indicate that bacteria involved in gut homeostasis and health (Actiobacteria [67] and Firmicutes Bacilli [68]), carbohydrate metabolism (Firmicutes and Firmicutes Clostridia [69]), and glucose homeostasis (Verrucomicrobia [70]) are positively correlated with long-standing pre-disposition to ME. These findings support other biological associations with ME. For example, Dupree and colleagues [14] in a case-controlled crossover trial, reported that changes in ME were associated with decreased Annexin A1, a mediator of the glucocorticoid, cortisol. This finding aligns with the current study’s findings because increased carbohydrate metabolism and improved glucose homeostasis were correlated with lower cortisol levels [60], which may influence Annexin A1 levels. Additionally, Boolani and colleagues (2019) [13] reported an association between ATP production and feelings of energy without differentiating between the physical and mental aspects of energy. Curiously, in the current study, trait PE was negatively correlated with Dorea and Holdemania, both of which have been correlated with high fiber diets [71], and lower levels of these have been correlated with higher blood pressure [72].

Trait MF was primarily correlated with Firmicutes Erysipelotrichi, bacteria primarily associated with TNF-α [73], a proinflammatory cytokine. These findings support the work by Loy and colleagues (2018) [12], who reported that proinflammatory cytokines are associated with feelings of fatigue only, without differentiating between the mental and physical aspects of fatigue. Findings from the current study further support the idea of inflammation being correlated with feelings of fatigue in that trait PF was correlated with Proteobacteria Gammaproteobacteria, which are reported to be higher in individuals who have inflammatory bowel disease [74,75,76]. Taken together, this study’s findings support the work by Loy and colleagues (2018) [12] in that fatigue was associated with inflammation.

Interestingly, *Anaerostipes* was correlated with trait ME, MF, and PF in the anticipated direction. Higher levels of *Anaerostipes* are associated with activation of fatty acid oxidation, synthesis, and lipolysis inhibition, which in turn decreases circulating lipid plasma levels and body weight [76]. It also suppresses colon inflammation and can downregulate insulin signal transduction in adipose tissue. Although this study did not differentiate which function the bacteria serve in each trait, based on previous findings [12] and the other bacteria associated with the traits, the authors hypothesize that *Anaerostipes’* anti-inflammatory function may be associated with MF and PF. In contrast, the metabolic function of the *Anaerostipes* bacteria may be correlated with trait ME.

When examining functional pathways, a majority were positively correlated with trait ME, while the functional pathway correlated with bacterial invasion of epithelial cells was negatively correlated with trait PE. Many of the functional pathways correlated with trait ME were metabolism-related pathways, suggesting that those who normally feel mentally energetic may produce gut substrates that impact metabolism in the gut and possibly the host. These findings may be important for nutrition science researchers because those reporting high trait ME may be hyper- or hypo-responders to some nutritional interventions (i.e., cocoa). Trait moods have been noted to influence acute responses to caffeine [23]; however, none of the pathways that were significant in the current study were correlated with methylxanthine metabolism.

The processed meats (frankfurters, sausages, corned beef, and luncheon meat that are made from beef, pork, or poultry) food group was the only one correlated with all four traits. These foods are also considered ultra-processed foods. Hall and colleagues [77] demonstrated in a randomized controlled trial that diets rich in ultra-processed foods promote excess calorie intake and weight gain. Limiting these foods may be an effective strategy to prevent obesity. Obese people often experience fatigue and decreased physical endurance [78]. The other identified foods groups in the current study were plant-based. As previously discussed, plants contain many substances that can be metabolized by the gut microbiota and impact host physiology. It is possible that the observed changes in the metabolome can be attributed to diet rather than trait because diet is known to alter the gut microbiome, which then leads to changes in the available small molecules, or nutrients, produced by the gut microbiota. Future studies should consider and account for the types of foods subjects eat.

### Limitations

As with all studies, this study is not without limitations. The primary limitation was the small sample size and the lack of significant findings when adjusting for multiple comparisons. However, since this was an exploratory study, and because this study was intended to be illustrative rather than definitive, the authors were unable to complete power and sample size calculations a priori. Nevertheless, these preliminary data show interesting findings, which can serve as foundational evidence for future investigations looking to determine the role of the gut microbiota in feelings of mental and physical energy and fatigue. Additionally, these study findings were limited to a single sample of healthy young adults and cannot be extrapolated to individuals who may have a diagnosis of diseases such as Inflammatory Bowel Syndrome (IBS), which has been known to influence gut microbiota composition. Future studies are recommended to add comparator subgroups with differing diagnoses so the relationships in this study can be compared by health status. Another potential limitation to this study is that stool samples and trait measures were collected two years apart. Future studies should consider collecting all microbiome and self-reported data from participants in closer temporal proximity. However, there is significant evidence that suggests that trait mental and physical energy and fatigue maintain temporal stability for as long as one year [15,16,47,79]. Additionally, there is scientific evidence that gut microbiota maintains temporal stability throughout adulthood [80,81]. However, exceptions are associated with inflammatory bowel syndrome [82,83], obesity [84,85], irritable bowel syndrome [86,87] (Jeffery et al., 2012; DuPont 2014), and type 2 diabetes [88] (Larsen et al., 2010). Therefore, participants who had any of these diseases/syndromes were excluded from the current study. Antibiotic therapy can also disrupt the gut microbiome, so individuals who had taken antibiotics less than three months prior to the study were also excluded. Fu et al. [89] examined the reliability and biological stability of fecal samples collected every six months for two years. They found that a single sample is sufficient to capture the majority of the variation in fecal microbiome from 16S rRNA gene sequencing. Multiple samples are needed for rare or less-abundant taxa, which are not reported in the current study. Finally, while 170 metabolites were identified in the fecal samples in this study; details were only provided for those that significantly differed in bivariate analyses and were included in subsequent analyses. While short-chain fatty acids were not measured for this study, future studies should examine these metabolites as related to trait mental and physical energy and fatigue.

## 5. Conclusions

The objective of this exploratory study was to identify potential correlations between gut microbiota and trait (long-standing pre-disposition to) mental and physical energy and fatigue, which can be used to guide future research. These findings provide evidence that the four traits (i.e., mental energy (ME), mental fatigue (MF), physical energy (PE), and physical fatigue (PF)) may have unique yet overlapping gut bacteria profiles. For example, the bacteria most often correlated with feelings of energy perform metabolic functions, while bacteria most often correlated with feelings of fatigue are associated with inflammation. This study suggests the need to explore the role of gut microbiota in understanding long-standing feelings of energy and fatigue among healthy young individuals.

## Figures and Tables

**Table 1:** Bacteria taxa significantly correlated with specific traits. Same colored bacteria names correlate with more than one trait.

	Correlation Coefficient	Significance
Trait Mental Energy		
p__Actinobacteria	0.469	0.037
p__Firmicutes	0.520	0.019
p__Firmicutes;c__Bacilli;o__Turicibacterales	0.470	0.037
p__Firmicutes;c__Bacilli;o__Turicibacterales;f__Turicibacteraceae	0.470	0.037
p__Firmicutes;c__Bacilli;o__Turicibacterales;f__Turicibacteraceae;g__Turicibacter	0.470	0.037
p__Firmicutes;c__Clostridia;o__Clostridiales;f__Lachnospiraceae;g__;s__	0.461	0.041
p__Firmicutes;c__Clostridia;o__Clostridiales;f__Lachnospiraceae;g__[Ruminococcus];s__gnavus	0.478	0.033
p__Firmicutes;c__Clostridia;o__Clostridiales;f__Lachnospiraceae;g__Anaerostipes;s__ *	0.480	0.032
p__Firmicutes;c__Clostridia;o__Clostridiales;f__Ruminococcaceae;g__;s__	0.454	0.044
p__Firmicutes;c__Clostridia;o__Clostridiales;f__Lachnospiraceae;g__Coprococcus;s__catus	0.479	0.032
p__Firmicutes;c__Clostridia;o__Clostridiales;f__Lachnospiraceae;g__Roseburia;s__faecis	0.558	0.011
p__Verrucomicrobia;c__Verrucomicrobiae	0.475	0.034
p__Verrucomicrobia;c__Verrucomicrobiae;o__Verrucomicrobiales	0.475	0.034
p__Verrucomicrobia;c__Verrucomicrobiae;o__Verrucomicrobiales;f__Verrucomicrobiaceae	0.475	0.034
p__Verrucomicrobia;c__Verrucomicrobiae;o__Verrucomicrobiales;f__Verrucomicrobiaceae; g__Akkermansia	0.475	0.034
Trait Mental Fatigue		
p__Firmicutes;c__Erysipelotrichi	0.451	0.046
p__Firmicutes;c__Erysipelotrichi;o__Erysipelotrichales	0.451	0.046
p__Firmicutes;c__Erysipelotrichi;o__Erysipelotrichales;f__Erysipelotrichaceae	0.451	0.046
p__Firmicutes;c__Clostridia;o__Clostridiales;f__Lachnospiraceae;g__Anaerostipes;s__	−0.532	0.016
Trait Physical Energy		
p__Firmicutes;c__Erysipelotrichi;o__Erysipelotrichales;f__Erysipelotrichaceae;g__Holdemania	−0.533	0.015
p__Firmicutes;c__Clostridia;o__Clostridiales;f__Lachnospiraceae;g__Dorea;s__	−0.463	0.040
p__Firmicutes;c__Clostridia;o__Clostridiales;f__Peptostreptococcaceae;g__;s__	−0.461	0.041
Trait Physical Fatigue		
p__Firmicutes;c__Clostridia;o__Clostridiales;f__Christensenellaceae;g__;s__	−0.630	0.003
p__Firmicutes;c__Clostridia;o__Clostridiales;f__Lachnospiraceae;g__Anaerostipes;s__	−0.448	0.048
p__Proteobacteria;c__Gammaproteobacteria;o__Pasteurellales	0.445	0.049
p__Proteobacteria;c__Gammaproteobacteria;o__Pasteurellales;f__Pasteurellaceae	0.445	0.049
p__Proteobacteria;c__Gammaproteobacteria;o__Pasteurellales;f__Pasteurellaceae;g__Haemophilus	0.512	0.021
p__Bacteroidetes;c__Bacteroidia;o__Bacteroidales;f__Bacteroidaceae;g__Bacteroides;s__	−0.451	0.046

* Bacteria highlighted in red was found in more than one trait.

**Table 2:** Predicted functional pathways significantly correlated with specific traits.

	Correlation Coefficient	Significance
Trait Mental Energy		
Cellular Processes; Cell Motility; Bacterial motility proteins	0.494	0.027
Genetic Information Processing; Replication and Repair; Non-homologous end-joining	0.523	0.018
Human Diseases; Infectious Diseases; African trypanosomiasis	0.501	0.025
Metabolism; Biosynthesis of Other Secondary Metabolites; Butirosin and neomycin biosynthesis	0.445	0.049
Metabolism; Biosynthesis of Other Secondary Metabolites; Flavonoid biosynthesis	0.531	0.016
Metabolism; Lipid Metabolism; Biosynthesis of unsaturated fatty acids	0.470	0.037
Metabolism; Metabolism of Terpenoids and Polyketides Biosynthesis of siderophore group nonribosomal peptides	0.450	0.046
Metabolism; Metabolism of Terpenoids and Polyketides; Carotenoid biosynthesis	0.621	0.003
Metabolism; Xenobiotics Biodegradation and Metabolism; Benzoate degradation	0.461	0.041
Metabolism; Xenobiotics Biodegradation and Metabolism; Chloroalkane and chloroalkene degradation	0.470	0.037
Metabolism; Xenobiotics Biodegradation and Metabolism; Dioxin degradation	0.464	0.039
Metabolism; Xenobiotics Biodegradation and Metabolism; Metabolism of xenobiotics by cytochrome P450	0.446	0.049
Metabolism; Xenobiotics Biodegradation and Metabolism; Naphthalene degradation	0.451	0.046
Metabolism; Xenobiotics Biodegradation and Metabolism; Xylene degradation	0.453	0.045
Organismal Systems; Digestive System; Carbohydrate digestion and absorption	0.511	0.021
Organismal Systems; Endocrine System; Insulin signaling pathway	0.447	0.048
Organismal Systems; Immune System; NOD-like receptor signaling pathway	0.446	0.049
Unclassified; Cellular Processes and Signaling; Electron transfer carriers	0.484	0.031
Unclassified; Metabolism; Lipid metabolism	0.448	0.048
Trait Physical Energy		
Human Diseases; Infectious Diseases; Bacterial invasion of epithelial cells	−0.604	0.005

**Table 3:** Correlation of traits with nutrients and food groups from 24 h recall.

		Trait Mental Energy	Trait Mental Fatigue	Trait Physical Energy	Trait Physical Fatigue
Folate, food (mcg)	Correlation	0.465 *	0.021	0.313	0.129
	Sig. (2-tailed)	0.039	0.931	0.178	0.588
Lycopene (mcg)	Correlation	−0.399	0.505	−0.438	0.503
	Sig. (2-tailed)	0.081	0.023	0.053	0.024
Total dark green, red and orange, starchy, and other vegetables; excludes legumes (cup eq.)	Correlation	0.500	−0.018	0.221	0.036
	Sig. (2-tailed)	0.025	0.940	0.350	0.880
Dark green vegetables (cup eq.)	P Correlation	0.456	0.052	0.322	0.187
	Sig. (2-tailed)	0.043	0.829	0.166	0.429
Grains defined as whole grains and which contain the entire grain kernel: bran, germ, and endosperm (oz. eq.)	Correlation	−0.609	0.383	−0.442	0.466
	Sig. (2-tailed)	0.004	0.095	0.051	0.038
Frankfurters, sausages, corned beef, and luncheon meat that are made from beef, pork, or poultry (oz. eq.)	Correlation	−0.790	0.538 *	−0.478	0.513
	Sig. (2-tailed)	<0.0001	0.014	0.033	0.021

* numbers highlighted in red are significantly different.

**Table 4:** Correlations between significant bacteria and significant nutrient/food groups.

Bacteria (All Belong to Firmicutes Phylum)		Folate, Food (mcg)	Lycopene (mcg)	Total Dark Green, Red and Orange, Starchy, and Other Vegetables; Excludes Legumes (Cup Eq.)	Dark Green Vegetables (Cup Eq.)	Grains Defined as Whole Grains and Which Contain the Entire Grain Kernel: Bran, Germ, and Endosperm (Oz. Eq.)	Frankfurters, Sausages, Corned Beef, and Luncheon Meat That Are Made from Beef, Pork, or Poultry (Oz. Eq.)
c__Clostridia;o__Clostridiales;f__Lachnospiraceae;g__Coprococcus;s__catus	Correlation	0.429	−0.354	0.391	0.491 *	0.209	−0.075
	Sig. (2-tailed)	0.059	0.126	0.088	0.028	0.376	0.753
c__Erysipelotrichi	Correlation	−0.281	0.470	−0.277	−0.212	0.344	0.262
	Sig. (2-tailed)	0.230	0.037	0.238	0.370	0.137	0.264
c__Erysipelotrichi;o__Erysipelotrichales	Correlation	−0.281	0.470	−0.277	−0.212	0.344	0.262
	Sig. (2-tailed)	0.230	0.037	0.238	0.370	0.137	0.264
c__Erysipelotrichi;o__Erysipelotrichales;f__Erysipelotrichaceae	Correlation	−0.281	0.470	−0.277	−0.212	0.344	0.262
	Sig. (2-tailed)	0.230	0.037	0.238	0.370	0.137	0.264
c__Erysipelotrichi;o__Erysipelotrichales;f__Erysipelotrichaceae;g__Holdemania	Correlation	−0.268	0.088	−0.339	−0.330	0.455	0.488
	Sig. (2-tailed)	0.254	0.713	0.143	0.155	0.044	0.029

* Numbers highlighted in red are significantly different.

## Data Availability

The data presented in this study are available on request from the corresponding author.

## Appendix A

**Table A1:** Subject information.

Variable	Median	Interquartile Range
Age (years)	31	7
Sex (Males, Females)	14, 6	
Weight (lbs)	180	54
Height (inches)	68	7
BMI (kg/m^2^)	25.3	5.1
Total Walking MET-min/week	1402.5	2883.4
Total Moderate MET-min/week total	1560.0	3532.5
Total Vigorous MET-min/week	2880.0	2520.0
Total Physical Activity MET-min/week	7939.5	5711.6
Sitting Total Minutes/week	2220.0	1245.0
Average Sitting Total Minutes/day	317.1	177.9
Trait Physical Energy	8.0	3.0
Trait Physical Fatigue	3.0	2.0
Trait Mental Energy	8.5	1
Trait Mental Fatigue	3.0	1.8

**Table A2:** Correlation of trait measures with four alpha diversity measures.

Alpha Diversity Measure		Trait Mental Energy	Trait Mental Fatigue	Trait Physical Energy	Trait Physical Fatigue
Evenness	Correlation Coefficient	0.099	−0.401	0.092	−0.387
	Significance	0.679	0.079	0.700	0.092
Shannon	Correlation Coefficient	0.293	−0.330	0.199	−0.432
	Significance	0.211	0.156	0.401	0.057
Observed Otus	Correlation Coefficient	0.432	−0.185	0.331	−0.390
	Significance	0.057	0.435	0.154	0.089
Faith PD	Correlation Coefficient	0.293	−0.367	0.247	−0.509
	Significance	0.209	0.111	0.294	0.022
